# Evidence of Rat Hepatitis E Virus Circulation through Wastewater Surveillance, Central Argentina

**DOI:** 10.3201/eid3201.251218

**Published:** 2026-01

**Authors:** Bianca Filoni, María Emilia Lucero, Guadalupe Di Cola, Anabella Fantilli, Alfonsina Roccia, Paola Sicilia, Liliana Luque, Ariana Cachi, María de los Ángeles Marinzalda, Gonzalo Castro, Gisela Masachessi, Viviana Ré, María Belén Pisano

**Affiliations:** Instituto de Virología Dr. J.M. Vanella, Universidad Nacional de Córdoba, Córdoba, Argentina (B. Filoni, M.E. Lucero, G. Di Cola, A. Fantilli, A. Roccia, L. Luque, A. Cachi, M. de los Ángeles Marinzalda, G. Castro, G. Masachessi, V. Ré, M.B. Pisano); Consejo Nacional de Investigaciones Científicas y Técnicas, Buenos Aires, Argentina (G. Di Cola, G. Masachessi, V. Ré, M.B. Pisano); Ministerio de Salud de la Provincia de Córdoba, Córdoba (P. Sicilia, L. Luque, G. Castro); Instituto Nacional de Medicina Aeronáutica y Espacial, Facultad de la Fuerza Aérea, Facultad de la Fuerza Aérea, Universidad de la Defensa Nacional, Córdoba (A. Cachi, M. de los Ángeles Marinzalda).

**Keywords:** Rat hepatitis E virus, R-HEV, *Rocahepevirus ratti*, wastewater-based epidemiology, viruses, zoonoses, enteric infections, Argentina

## Abstract

During 2023–2024, we detected rat hepatitis E virus in 67.7% of wastewater samples from central Argentina. This high level of detection opens new inquiries in the region, highlighting the need to investigate the virus in both animal reservoirs and humans, with a focus on hepatitis cases of unknown etiology.

Rat hepatitis E virus (R-HEV) (family Hepeviridae, species *Rocahepevirus ratti*) is an emerging cause of viral hepatitis in humans that belongs to the Hepeviridae family, the same family as hepatitis E virus (HEV) (species *Paslahepevirus balayani*), a major cause of hepatitis in humans (*1*). Rats are the primary reservoirs of R-HEV, and the virus has been detected in several countries, mainly in Europe, and in different species of rodents (*2*). Since the first reported human infection in a transplant patient in 2018 in Hong Kong, China, reports of human cases in immunosuppressed and immunocompetent patients from Asia, Europe, and North America have been increasing (*3*–*5*). Those cases highlight the zoonotic potential of R-HEV, positioning this agent as a growing concern for public health (*6*).

R-HEV and HEV are transmitted by the fecal–oral route. The virus is shed in the stool of infected humans and animals and subsequently discharged into wastewater. R-HEV can be studied under the One Health approach, enabling us to consider the interactions between environment, hosts, and the virus (*7*). Wastewater surveillance has proven to be effective in detecting the emergence of both well-known and little-studied enteric viruses in different regions and in tracking their spread and circulation within various communities (*1*,*8*). In South America, we found only 1 recent report of R-HEV detection from naturally growing mangrove bivalve mollusks collected for local sale in a touristic area of Brazil and demonstrating a low detection rate (2.2%) (*9*). Because of the scarce evidence of R-HEV circulation in South America, we aimed to assess the presence of R-HEV in wastewater from Córdoba, a central province of Argentina, as an indicator of viral circulation in the region.

## The Study

**Figure 1:**
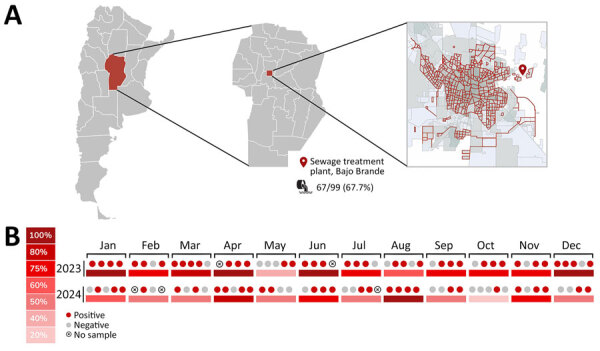
Emerging rat hepatitis E virus detection in Argentina, 2023–2024. A) Study area in the province of Córdoba (red shading), located in the central region of Argentina, and the location of Bajo Grande wastewater treatment plant in the city of Córdoba. Red lines indicate the sewage network, which covers 56.9% of the city’s population. B) Frequency of detection of rat hepatitis E virus during 2023–2024, per month. The circles indicate detections per week sampled within each month.

During January 2023–December 2024, we collected sewage samples weekly (total n = 99: 2023, n = 49; 2024, n = 50) from the Bajo Grande wastewater treatment plant (BG-WWTP) of Córdoba city, the capital of the province of Córdoba, with 1,505,250 inhabitants (Figure 1, panel A). BG-WWTP receives the waste of ≈56.9% of the city’s population (https://censo.gob.ar/index.php/mapa_desague_red_publica2). We concentrated each 500-mL sample 100 times by using the polyethylene glycol-6000 precipitation method, as described previously (*8*), and conducted RNA extraction by using the Nucleic Acid Extraction Versatile Spin Kit (Anatolia Geneworks, https://www.anatoliageneworks.com). We conducted molecular detection of the virus by using a real-time reverse transcription PCR (RT-PCR) targeting a 69-bp fragment of the open reading frame 1 genomic region, as described previously (*10*). We used the TaqMan Fast Virus 1-Step master mix (Thermo Fisher Scientific, https://www.thermofisher.com), performed in a StepOne Real-Time PCR (Thermo Fisher Scientific). For positive controls we used synthetic oligonucleotide (Appendix), and for negative controls we used sterile water. We considered samples RNA R-HEV positive if we observed an S-shaped curve in the specific detection channel of each specimen and obtained a cycle threshold value <40 for viral specific target, as previously described (*1*,*8*). With the aim of genetic characterization of positive samples, we conducted heminested RT-PCR amplification of a 338-bp fragment within the open reading frame 1 genomic region of the Hepeviridae family (*11*). We purified PCR products by using the PureLink Quick Gel Extraction Kit (Invitrogen, https://www.invitrogen.com) and sequenced in both directions by using an Applied Biosystem 3500XL Genetic Analyzer (Thermo Fisher Scientific). We conducted phylogenetic analyses by using MEGA version11 (*12*), IQ-Tree (http://iqtree.cibiv.univie.ac.at), and FigTree (https://tree.bio.ed.ac.uk/software/figtree). In addition, we used a newly developed genotyping tool for R-HEV classification (*13*) and constructed an identity matrix to determine the similarity between the sequences obtained by using BioEdit software version 7.7.1 (https://bioedit.software.informer.com/Descargar-gratis).

**Figure 2:**
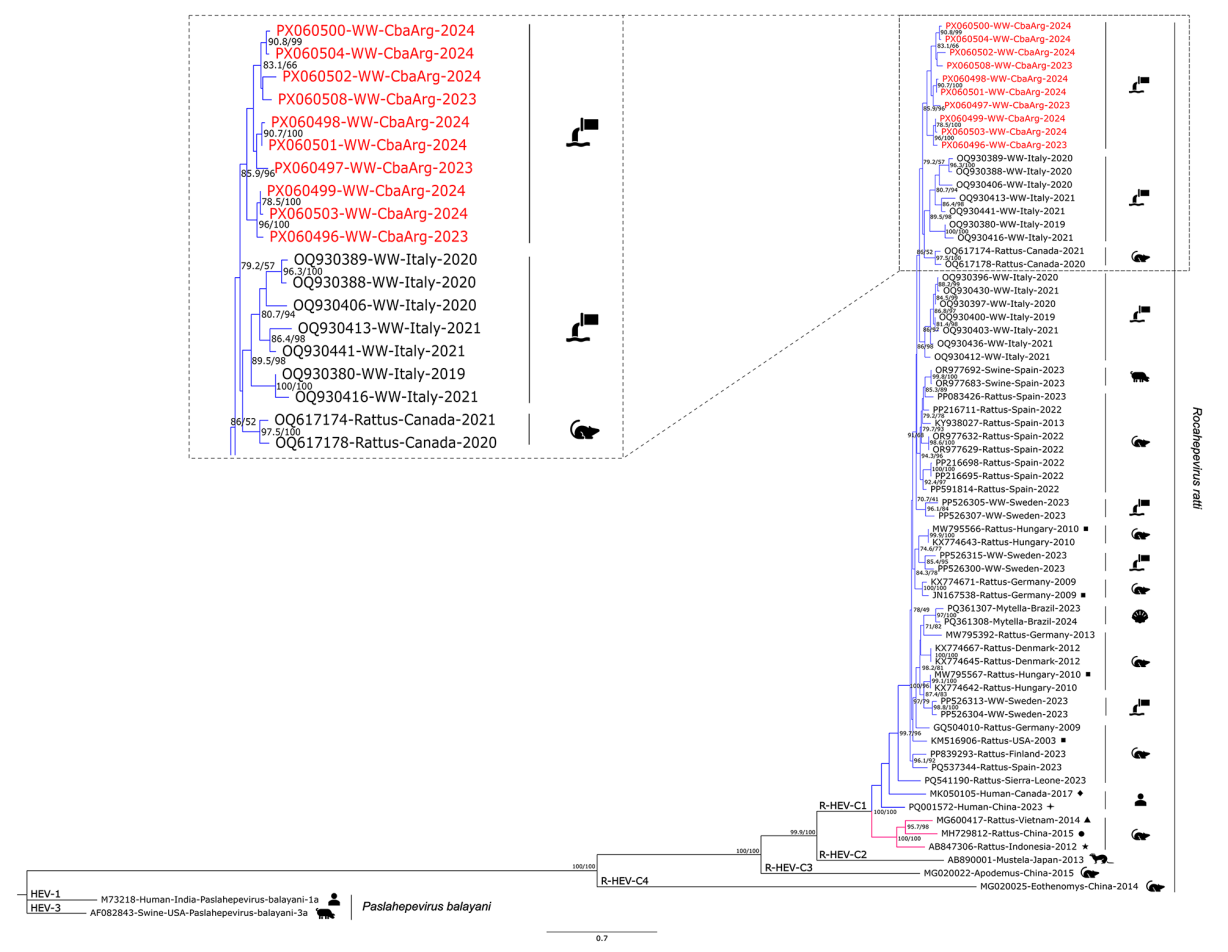
Maximum-likelihood phylogenetic tree on the basis of a 338 bp fragment of the open reading frame 1 genomic region of rat hepatitis E virus (R-HEV; *Rocahepevirus ratti*) from Argentina, 2023–2024. Red text indicates sequences obtained during this study. The tree includes representative sequences of each R-HEV genotype available in GenBank database, additional sequences of proposed clades (clade I in blue, clade II in pink) and subtypes a (square), b (circle), c (5-pointed star), d (4-pointed star), e (rhombus), and f (triangle) (*13*). Two hepatitis E virus (*Paslahepevirus balayani*) sequences (genotypes 1 and 3) were used as outgroups. Enlarged area shows sequences from this study and the first 10 most similar sequences from BLAST analysis (https://blast.ncbi.nlm.nih.gov). Statistical support values are indicated at nodes; only supports over 70/70 are shown. Scale bar represents the number of substitutions per site.

Of the 99 samples analyzed, 67.7% (95% CI 58.5%–76.9%; n = 67) tested positive for R-HEV detection by RT-PCR (Appendix Table 1). By year, 77.6% ; (95% CI 63.0%–87.8%; n = 38) of samples from 2023 were positive, and 58.0% (95% CI 43.3%–71.5%; n = 29) of samples from 2024 were positive (Figure 1, panel B). Of the 67 positive samples by real-time RT-PCR, 14 were positive by the heminested RT-PCR, and 10 were sequenced. The phylogenetic tree confirmed the virus species identity as *Rocahepevirus ratti* (GenBank accession nos. PX060496–504, PX060508) and showed grouping with sequences from sewage samples from Italy and rodents from Canada, within genotype C1 (Figure 2). The genotyping tool indicated that 9 sequences from this study belonged to the proposed clade I, subtype a. One sequence yielded an indeterminate result (Appendix Table 2). The similarity between the obtained sequences varied from 0.71 to 0.98 (Appendix Table 3).

## Conclusions

This study revealed R-HEV circulation in Argentina, confirming the presence of emerging R-HEV in South America. R-HEV detection in wastewater samples shows a high level of circulation (high detection rate) in the human environment from the central region of the country. The low number of sequenced samples could be explained by the dilution and degradation of viral RNA within the wastewater matrix, driven by factors such as pH fluctuations, temperature variation, and complex biochemical interactions. Those conditions might promote the formation of short, partially degraded RNA fragments that remain detectable by real-time RT-PCR but are insufficient for amplifying longer genomic regions required for conventional PCR and sequencing. Similar patterns were reported in previous wastewater studies (*8*). Contamination of wastewater by human waste is a possible source of the virus, although the involvement of rodents, the natural viral host, in sewers is the most likely scenario (*14*). To date, we have not found evidence of human or animal infections in Argentina; therefore, our results open new and unexplored fields for R-HEV research in South America. Studies from the past 2 years have demonstrated the presence of R-HEV RNA in wastewater from countries in Europe, underscoring the value of wastewater-based surveillance for detecting emerging viruses such as R-HEV (*1*,*8*). Concomitantly, studies were conducted to clarify the clinical implications of R-HEV in Europe. Those studies revealed that R-HEV could cause symptomatic disease in humans, both in immunosuppressed and immunocompetent patients, producing mild illness, severe hepatitis, and potentially death (*4*).

Phylogenetic analysis revealed that the sequences obtained in our study clustered together, although they were not identical (similarity 0.71–0.98). That clustering could suggest that the same strains circulated over the 2-year period. The sequences grouped close to strains previously detected in sewers in Italy and in rodents from Canada within genotype C1 (*1*,*15*). However, because of the limited available data—very few sequences of this virus are currently deposited in GenBank, and 3 are from South America—and because of the relatively short genomic fragment analyzed, definitive conclusions cannot be drawn. Recently published studies suggest our sequences would be assigned to clade I, subtype a (*13*).

In line with the One Health approach, our findings reinforce the need to further investigate R-HEV circulation in Argentina and South America. This effort should integrate environmental surveillance with clinical research, such as testing samples from symptomatic patients for R-HEV RNA and exploring potential viral reservoirs such as rats. In addition to rodents, attention should be given to other possible hosts, such as pigs, in which R-HEV infections have been documented (*6*).

Appendix Additional information about evidence of rat hepatitis E virus circulation through wastewater surveillance, central Argentina.
