# Supplementary material for: Evidence of Rat Hepatitis E Virus Circulation through Wastewater Surveillance, Central Argentina
Source: Emerg Infect Dis. 2026 Jan;32(1):133–6. doi: 10.3201/eid3201.251218 (PMC12870092; doi:10.3201/eid3201.251218)
Supplement: Appendix — Additional information about evidence of rat hepatitis E virus circulation through wastewater surveillance, central Argentina. [file 25-1218-Techapp-s1.pdf]

Article DOI: https://doi.org/10.3201/eid3201.251218

EID cannot ensure accessibility for supplementary materials supplied by authors. Readers who have difficulty accessing supplementary content should contact the authors for assistance.

Evidence of Rat Hepatitis E Virus Circulation through Wastewater Surveillance, Central Argentina

Appendix

Sequences of positive controls

This appendix contains the nucleotide sequences of the positive controls used in the PCR assays conducted in this study.

Sequence of R-HEV positive control used in real-time RT-PCR assay (5′–3′) (*Rocahepevirus ratti*):

CTTGTTGAGCTTTTCTCCCCTTTGCAGCTTGTCTTTGAGCCCGCTGTTTCTTGG TCGCATCCGGTACAG

Sequence of positive control used for RT-heminested PCR (5′–3′) (*Paslahepevirus balayani*):

ACYTTTTGTGCTCTGTTTGGTCCGTGGTTCCGTGCCATTGAAAAAGAAATATT AGCCCTGCTCCCSCCTAACATCTTTTATGGCGACGCTTATGAGGAGTCGGTGTTTGCC GCCGCTGTGTCTGGGGCAGGGTCTTGCATGGTGTTCGAAAATGATTTCTCGGAGTTT GACAGCACCCAGAATAATTTCTCTCTTGGCCTTGAGTGTGTGGTTATGGAAGAGTGC GGCATGCCTCAATGGCTAATCAGGTTGTATCACCTGGTCCGGTCAGCTTGGATTTTG CAGGCGCCGAAAGAGTCTCTTAAAGGTTTTTGGA

Appendix Table 1. Cycle threshold (Ct) values and sampling dates of R-HEV–positive samples obtained by real time RT-PCR assay.

Sample ID	Sampling Date (day/month/year)	Ct value (R-HEV real time RT-PCR)
1967	03/01/2023	33,5
1970	10/01/2023	35,5
1973	17/01/2023	32,1
1986	24/01/2023	34,0
1972	01/02/2023	32,1
1966	09/02/2023	33,4
1975	22/02/2023	32,1
2041	01/03/2023	32,5
2070	07/03/2023	34,7
2071	15/03/2023	33,6
2089	21/03/2023	32,4
2101	10/04/2023	32,2
2105	17/04/2023	31,8
2250	25/04/2023	33,9
1	22/05/2023	34,1
2	31/05/2023	33,7
3	07/06/2023	35,1
4	12/06/2023	32,3
5	22/06/2023	33,6
6	04/07/2023	32,9
7	14/07/2023	33,8
8	21/07/2023	33,5
11	10/08/2023	33,4
12	14/08/2023	37,8
14	29/08/2023	38,0
16	13/09/2023	33,6
17	20/09/2023	32,3
19	28/09/2023	32,1
20	10/10/2023	33,7
21	19/10/2023	33,4
22	26/10/2023	33,0
23	01/11/2023	34,4
25	13/11/2024	33,0
26	27/11/2023	35,0
27	01/12/2023	37,5
28	07/12/2023	34,4
29	11/12/2023	32,7
30	26/12/2023	34,1
33	08/01/2024	35,0
35	24/01/2024	32,5
36	31/01/2024	32,7
37	12/02/2024	33,5
39	08/03/2024	32,5
41	18/03/2024	32,8
43	04/04/2024	37,0
44	11/04/2024	33,6
46	24/04/2024	34,9
47	30/04/2024	34,4
48	07/05/2024	34,5
49	14/05/2024	33,7
53	11/06/2024	35,7
54	18/06/2024	33,2
55	26/06/2024	34,2
59	18/07/2024	33,9
60	22/07/2024	34,0
61	06/08/2024	35,2
62	12/08/2024	31,9
63	19/08/2024	35,1
64	26/08/2024	35,4
67	20/09/2024	36,6
68	23/09/2024	34,0
72	21/10/2024	31,7
74	04/11/2024	34,6
76	22/11/2024	34,9
77	26/11/2024	35,2
80	20/12/2024	33,4
81	27/12/2024	36,0

Appendix Table 2. Subtyping results of R-HEV sequences obtained with the R-HEV Subtyping Tool (https://rhev-subtyping.streamlit.app).

Sample	Closest Reference by P-Distance (value)	Closest Reference by ML Patristic Distance (value)	Clade Assignment	Subtype Assignment
PX060496	KM516906.1 (0.1577)	JN167537.1 (0.2320)	I	a
PX060508	PQ488559 (0.1327)	OP947207.1 (0.2194)	I	a
PX060497	KM516906.1 (0.1283)	NC_038504.1 (0.1879)	Not-determined	Not-determined
PX060499	JN167538.1 (0.1205)	JN167537.1 (0.1653)	I	a
PX060500	MW795566.1 (0.1430)	JN167537.1 (0.1719)	I	a
PZ060501	MW795567.1 (0.1278)	MW795567.1 (0.1492)	I	a
PX060498	MW795567.1 (0.1414)	MW795567.1 (0.1788)	I	a
PX060502	PQ488559 (0.1311)	OM037395.1 (0.1587)	I	a
PX060503	JN167538.1 (0.1267)	JN167537.1 (0.1645)	I	a
PX060504	PQ488559 (0.1343)	JN167537.1 (0.1864)	I	a

Appendix Table 3. R-HEV sequence identity matrix performed with the software Bioedit v.7.7.1, including sequences from this study and those most similar from GenBank.

Seq->	OQ617174	OQ617178	OQ930380	OQ930388	OQ930389	OQ930406	OQ930413	OQ930416	OQ930441	PX060496	PX060497	PX060498	PX060499	PX060500	PX060501	PX060502	PX060503	PX060504	PX060508
OQ617174	ID	0,939	0,792	0,796	0,788	0,788	0,803	0,803	0,83	0,826	0,742	0,8	0,837	0,771	0,811	0,826	0,83	0,815	0,826
OQ617178	0,939	ID	0,8	0,796	0,781	0,788	0,803	0,796	0,822	0,807	0,734	0,792	0,811	0,779	0,803	0,833	0,803	0,822	0,849
OQ930380	0,792	0,8	ID	0,849	0,86	0,86	0,845	0,954	0,856	0,852	0,679	0,856	0,864	0,713	0,86	0,875	0,86	0,86	0,886
OQ930388	0,796	0,796	0,849	ID	0,966	0,886	0,845	0,849	0,879	0,845	0,649	0,822	0,856	0,72	0,833	0,852	0,852	0,867	0,867
OQ930389	0,788	0,781	0,86	0,966	ID	0,883	0,845	0,845	0,886	0,841	0,66	0,837	0,867	0,728	0,849	0,86	0,867	0,875	0,86
OQ930406	0,788	0,788	0,86	0,886	0,883	ID	0,845	0,852	0,875	0,852	0,667	0,852	0,856	0,713	0,864	0,879	0,852	0,871	0,879
OQ930413	0,803	0,803	0,845	0,845	0,845	0,845	ID	0,837	0,916	0,864	0,664	0,852	0,867	0,69	0,856	0,852	0,856	0,837	0,845
OQ930416	0,803	0,796	0,954	0,849	0,845	0,852	0,837	ID	0,867	0,845	0,667	0,83	0,864	0,694	0,841	0,849	0,86	0,845	0,879
OQ930441	0,83	0,822	0,856	0,879	0,886	0,875	0,916	0,867	ID	0,867	0,675	0,852	0,867	0,709	0,864	0,852	0,864	0,867	0,86
PX060496	0,826	0,807	0,852	0,845	0,841	0,852	0,864	0,845	0,867	ID	0,732	0,901	0,966	0,739	0,916	0,871	0,954	0,886	0,871
PX060497	0,742	0,734	0,679	0,649	0,66	0,667	0,664	0,667	0,675	0,732	ID	0,747	0,732	0,877	0,758	0,713	0,728	0,735	0,724
PX060498	0,8	0,792	0,856	0,822	0,837	0,852	0,852	0,83	0,852	0,901	0,747	ID	0,901	0,762	0,984	0,89	0,898	0,92	0,905
PX060499	0,837	0,811	0,864	0,856	0,867	0,856	0,867	0,864	0,867	0,966	0,732	0,901	ID	0,75	0,916	0,886	0,988	0,894	0,886
PX060500	0,771	0,779	0,713	0,72	0,728	0,713	0,69	0,694	0,709	0,739	0,877	0,762	0,75	ID	0,773	0,777	0,747	0,815	0,777
PX060501	0,811	0,803	0,86	0,833	0,849	0,864	0,856	0,841	0,864	0,916	0,758	0,984	0,916	0,773	ID	0,901	0,913	0,932	0,916
PX060502	0,826	0,833	0,875	0,852	0,86	0,879	0,852	0,849	0,852	0,871	0,713	0,89	0,886	0,777	0,901	ID	0,875	0,924	0,924
PX060503	0,83	0,803	0,86	0,852	0,867	0,852	0,856	0,86	0,864	0,954	0,728	0,898	0,988	0,747	0,913	0,875	ID	0,89	0,875
PX060504	0,815	0,822	0,86	0,867	0,875	0,871	0,837	0,845	0,867	0,886	0,735	0,92	0,894	0,815	0,932	0,924	0,89	ID	0,947
PX060508	0,826	0,849	0,886	0,867	0,86	0,879	0,845	0,879	0,86	0,871	0,724	0,905	0,886	0,777	0,916	0,924	0,875	0,947	ID
